# Extra Molting and Selection on Nymphal Growth in the Desert Locust

**DOI:** 10.1371/journal.pone.0155736

**Published:** 2016-05-26

**Authors:** Benjamin Pélissié, Cyril Piou, Hélène Jourdan-Pineau, Christine Pagès, Laurence Blondin, Marie-Pierre Chapuis

**Affiliations:** 1 CIRAD, UMR CBGP, F-34398, Montpellier, France; 2 CIRAD, UPR B-AMR, F-34398, Montpellier, France; University of Rouen, France, FRANCE

## Abstract

In insects, extra-molting has been viewed as a compensatory mechanism for nymphal growth that contributes to optimize body weight for successful reproduction. However, little is known on the capacity of extra-molting to evolve in natural populations, which limits our understanding of how selection acts on nymphal growth. We used a multi-generational pedigree, individual monitoring and quantitative genetics models to investigate the evolution of extra-molting and its impact on nymphal growth in a solitarious population of the desert locust, *Schistocerca gregaria*. Growth compensation via extra-molting was observed for 46% of the females, whose adult weight exceeded by 4% that of other females, at a cost of a 22% longer development time. We found a null heritability for body weight threshold only, and the highest and a strongly female-biased heritability for extra molting. Our genetic estimates show that (1) directional selection can act on growth rate, development time and extra-molting to optimize body weight threshold, the target of stabilizing selection, (2) extra-molting can evolve in natural populations, and (3) a genetic conflict, due to sexually antagonistic selection on extra-molting, might prevent its fixation. Finally, we discuss how antagonistic selection between solitarious and gregarious environments and/or genetic correlations between growth and phase traits might also impact the evolution of extra-molting in locusts.

## Introduction

Adult body size is a crucial quantitative life-history trait, closely related to individual fitness [1–5]. The body size threshold is especially thought to be under strong selection as it determines the minimum size individuals need to attain for successful reproduction [5]. In species in which growth only occurs during juvenile development, the optimal size for reproductive success may be determined before sexual maturity, at the transition to the adult life history stage. Different values of adult body size can be attained by altering growth rate and/or development time [6], both strategies with their own costs and constraints and not mutually exclusive [5,7]. Although selection should always optimize body size, life histories can also be time-constrained, for example in seasonal environments where organisms may have a limited time to reach their adult body size threshold [8].

In insects, overall developmental plasticity is limited by the fact that intra-instar mass increment is physiologically constrained [9]. Some species have the ability to modulate nymphal growth by adding (or skipping) one or several instar(s) [6,10,11]. Adding an instar (thereafter 'extra molting') is usually viewed as a catch-up growth strategy allowing for instance small-size offspring or individuals raised on low-quality food to increase their body size at adult emergence [10,12], though at the cost of a longer development time and potentially lower survival rates [13]. Seasonal climatic conditions (e.g. temperature variations; [14,15]) are likely to impact individuals' physiological conditions, on which depends their probability to add an extra molt. In any case, the entire developmental pattern can be modified by extra molting, which represents a major mechanism of phenotypic plasticity in insect development.

Extra molting also plays a role in the development of sexual size dimorphism (SSD) [6] since the selection regime is usually different in the two sexes. Usually, females grow to larger sizes than males (which develop faster), leading to the so-called female-biased SSD [11,16]. Such divergent selection pressures between sexes, or sexually antagonistic (SA) selection, is common in natural populations. SA selection generates intralocus sexual conflict because of shared genetic structure. This sexual conflict can be resolved through the evolution of sexual dimorphism in the presence of significant additive genetic variance, though it may often persist with severe costs in terms of fitness [17]. Under SA selection, additive genetic variance can differ in males and females due to sex-specific differences in allele frequencies (due to sex chromosome linkage) or in allele expression [18]. Indeed, traits showing sexual dimorphism often have greater additive genetic variances in the sex in which it is selected for [18,19].

Extra molting is intimately linked to growth rate, adult body weight threshold and development time. However, to our knowledge, the few studies investigating extra molting in insects did not explore its potential to evolve, *via* estimates of genetic variances and correlations. Therefore, we still do not know how extra molting responds to selection and how its evolution is linked to that of other growth traits [6], even though it is of prime importance to understand how selection shapes and acts on the evolution of nymphal development in natural populations [20]. Life-history traits that are intimately linked to fitness should be under strong stabilizing selection that is likely to reduce the levels of genetic variance. On the contrary, genetic variance of secondary traits should be maintained to some degree, allowing directional selection to act on them [20–22]. One may thus expect body weight threshold at adult emergence (as the main target of nymphal development) to exhibit low heritability and evolvability values, while extra molting (as an adjustment factor of nymphal development) should display large genetic variance estimates.

The desert locust, *Schistocerca gregaria* Forsk., provides a unique study system for exploring extra molting and selection on nymphal growth. First, our current understanding of the physiological mechanisms and evolutionary determinants of instar number is mainly based on holometabolous insects, in which the extra-molt event occurs at the last larval instar (e.g. the lepidopteran *Manduca sexta*; [23]). In contrast, the desert locust is a hemimetabolous insect, in which the 'decision' to undergo further growth through an additional instar occurs at the beginning of immature development, such as for the field grasshopper *Chorthippus brunneus*, whose development is extensively studied [24,25]. In *S*. *gregaria*, the extra instar takes place before the reversal of the wing rudiments, which occurs after the 3^rd^ instar (or extra-3^rd^) and before the 4^th^ instar in the nymphs developing in five (or six) instars, respectively [26,27]. The two types of nymphs can be discriminated from their 3rd chronological stage based on female genitalia and wingpads [28]. In addition, some hormones in locusts are unknown in holometabolous insects (e.g. [29,30]), though our knowledge of molt-triggering hormones in hemimetabolous insects is still fragmentary [31].

Moreover, in this species, extra molting is not only more frequent in females [32,33] and induced by low-quality food [33], it is also phase-dependent. Phase polyphenism is a process of density-dependent changes of various morphological, behavioral, developmental and reproductive traits [33–36]. In *S*. *gregaria*, gregarious individuals (from dense populations) mostly go through 5 molts, while solitarious individuals (in low-density populations) tend to undergo an extra molt, leading to 6 nymphal molts [27,33,37,38]. Because solitarious locusts produce significantly smaller hatchlings than gregarious ones [33,39,40], the catch-up growth strategy has been considered as the main explanation for extra molting in solitarious populations of the desert locust [28,32,41]. Interestingly, the two main types of hormone involved in nymphal molts, juvenile hormones (JH) and ecdysones, are known (1) to be regulated differently in solitarious and gregarious nymphs and (2) to participate (among others) in the determination of the phase states. For example, JH haemolymph titres are higher in scattered (solitarious) nymphs than in crowded (gregarious) relatives [42] and are also known to be involved in the induction of green body color and brachyptery, and in the repression of cuticle melanization [42–45], all characteristics typical of the solitarious phase state [46–48].

In this study, we describe the pattern of nymphal growth in solitarious desert locusts. We quantify the relative effect of extra molting, sex and hatching on nymphal growth rate, development time and maximal weight in a natural population raised in environmentally-controlled, optimal laboratory conditions. We also investigate the genetic parameters of these traits by using a 2-generational pedigree to identify which trait involved in nymphal development might be able to respond to selection in *S*. *gregaria*. Finally, we discuss the evolution of extra molting in the context of antagonistic selection on nymphal development between sexes and phases.

## Materials and Methods

### Study population and rearing conditions

The locusts we measured came from a second generation lab population (G_2_). In December 2010, 57 G_0_ fertilized females were sampled from a wild population located in Mauritania (Yaghref, region of Akjoujt) by the National Anti-locust Center, with a density of 10,000 individuals/ha. No specific permissions were required for the collection of female *Schistocerca gregaria* in Mauritania. Although *Schistocerca gregaria* is not a protected or endangered species, original samplings were conducted by the national authority in charge of controlling this pest species: the "Centre National de Lutte Antiacridienne" (CNLA—National Anti-locust Center) of Mauritania (www.cnla.mr). The sampled females were introduced in 8 m^3^ (2x2x2m) field cages until they laid eggs (G_1_). After reception in our laboratory, 9 egg pods were maintained at 31°C in small cages (20x20x25 cm; one pod per cage), the emerging offspring remaining with their siblings until the second molt. These 101 G_1_ laboratory individuals were individually transferred in 1L plastic boxes with a pierced lid when they reached their third nymphal stage. Isolated rearing facilities were the same as those described in [49], which have been proved to efficiently induce solitarious phase locusts. Accordingly, G_1_ adults displayed the typical characteristics of solitary insects, as shown by the ratio of average hind femur length to head capsule width of 3.81 [33,46,50]. We then created 37 male-female non-sib pairs of sexually mature adults (i.e. 6 to 8 weeks after adult emergence) by introducing each female inside a male’s box. Pairs were left together during 3 days, after which the females were returned for the following 4 days to their original box, with access to a laying tank containing sterilized sand with 10% water. This 7-day protocol was repeated until production of an egg pod, immediately incubated at 34°C, which is the optimal temperature for nymphal development [33]. Fourteen G_1_ male-female pairs produced at least one egg pod and gave rise to viable offspring. From each pod, 10 G_2_ full-sibs (5 males and 5 females) were randomly selected directly after hatching (i.e. within the first 12 hours), weighed to the nearest milligram and transferred into individual plastic boxes for the rest of their development. Laboratory G_1_ and G_2_ individuals were maintained at 50–60% humidity, under a 12h:12h photoperiod and fed ad libitum with fresh wheat shoots and bran.

### Pedigree and quantitative genetics design

Common parentage of G_1_ individuals were uncertain since (i) females could have laid more than one egg pod and (ii) in the field *S*. *gregaria* females may copulate with multiple males [51]. We therefore inferred G_1_ relationships based on multi-locus genotyping and a maximum-likelihood method. We genotyped 10 microsatellite loci as described in [52] (i.e. SgM40, SgM41, SgM51, SgM66, SgM74, SgM86, SgM87, SgM88, SgM92, SgM96). We used the software Colony v2 [53] to reconstruct relationships between individuals while accounting for typing errors and mutations (set to a rate of 0.02). Allele frequency estimates were refined by accounting for the estimated relationships in a sample. Our analysis showed the presence of half-sibs within four egg-pods obtained from the field cages, for a total of 22 distinctive G_0_ genotypes. We then constructed a 2-generation pedigree matrix to be used in quantitative genetics analyses. From the 22 identified G_0_ genotypes, 10 were involved in the production of the 28 G_1_ parents used to produce the G_2_ we monitored. These analyses also revealed that genetic variation was still substantial, with an average of 13 wild alleles per microsatellite locus recovered in the G_1_ laboratory strain.

### Nymphal growth survey

We monitored the development of 140 G_2_ individuals (14 families of 10 full-sibs each) from the moment they hatched. We ceased our measurements at adult emergence rather than at sexual maturity since there is no phenotypic character to assess the maturation state of isolated solitarious individuals in the desert locust. Each day, we weighed the insects and recorded any molting (or death) event. Individuals that underwent an extra molt were identified unambiguously, since nymphal stages and exuviae are easily identifiable. We also confirmed the identity of extra molting individuals at the adult stage, since in the subfamily Cyrtacanthacridinae each molting event leaves a dark stripe on the eye, the number of which correlates strictly with the number of nymphal instars the individual has been through [27,38,54,55].

### Studied traits

We studied four intimately linked life-history traits depicting nymphal growth: growth rate, maximal nymphal weight, development time and extra molting. Growth rate was calculated within each nymphal instar as the coefficient of the log-linear regression of body weight on age (in days) from molting (or hatching for the first instar) to the day at maximal instar weight. Development time was the age (in days) at which an individual reaches its adult emergence. Molt strategy was recorded as a binary indicator: 0 = normal or 1 = extra molting. We assessed the maximal nymphal weight rather than the weight at adult emergence since the fifth instar nymph/young fledged adult ceases to feed and looses weight 24-48h before/after imaginal molt [56]. We also studied the probability of survival at each development stadia (nymphal stadia, first adult week).

### Statistical analyses

#### Explanatory factors

In the statistical analyses, we used sex and extra molting as main factors affecting nymphal growth. We also added hatchling body weight (hatch weight) as a covariate to the analyses of growth characters, in order to account for genetic variances potentially erroneously inflated by some maternal effects in our full-sibs design. For example, large females produce eggs with more yolk than small females, and therefore their offspring grow faster [57], which adds a non-genetic component to the estimated covariance among full sibs. Finally, we corrected for crippling events prior to further analyses, as it could bias our estimates and analyses of growth rate and maximal nymphal weight. Crippling is a frequent strategy that occurs when an individual sacrifices one of its hind legs due to stress when attacked by a natural enemy or, in our case, manipulated during the measuring procedure. On an independent sample of solitarious *S*. *gregaria*, we estimated that a hind leg represents 7.3±0.6% and 6.8±0.8% of the entire body weight for males and females respectively. For each crippled adult, we then identified the day at which crippling occurred in its development and added the corresponding missing fraction of body weight for every recorded day until adult emergence.

#### Survival

We tested for the effect of sex and extra molting on three types of survival probabilities: between successive nymphal instars (ignoring the extra-molt event taking place between the 3^rd^ and 4^th^ nymphal stadia), during the first seven days of adult life, and between the emergence of the 4^th^ instar and the 7^th^ day of adult life (i.e. after the extra-molt event). We fitted 7 full generalized linear models (GLMs; one per nymphal stadium, one for the whole nymphal period and one for the first week of adult life) assuming a binomial distribution of the data and a full factorial design. We ranked all possible models from null to full (i.e., including all variables as well as every simple interaction) based on their AIC scores and selected the model with the lowest AIC. Final models were checked for normality and homoscedasticity of residuals.

#### Nymphal development

We tested the effect of hatch weight, sex, molt strategy and every simple interaction between pairs of factors on growth rate, maximal nymphal weight and development time. For maximal nymphal weight and development time, we ran every possible univariate linear models (i.e. including the 3 fixed effects and the 3 simple interactions). For growth rate, we used a linear mixed model including hatch weight, sex, molt strategy and nymphal instar as fixed effects and identity of the individual as a random effect (accounting for the repeated measurements at each nymphal instar). Variable selection was done the same way as in survival analyses (see above the sub-section ‘Survival‘). Nevertheless, we also considered models displaying a ΔAIC (difference of AIC to the lowest AIC score) lower than 2, which is justified by the construction of AIC itself [58,59].

Since the potential insertion of an extra molt is determined between the 2^nd^ and 3^rd^ instars [60], the minimal body size needed for an individual to develop normally until adulthood without adding an extra molt was calculated at the 2^nd^ nymphal stage. To this aim, we derived for each sex the maximal body weight within the 2^nd^ stadium under which the probability of an individual to add an extra molt exceeded 95% from predictions of a logistic regression model [14,61]. This threshold body weight predicts the ultimate number of instars for an individual.

#### Genetic parameters of life-history traits

For each trait analysed, we applied univariate animal models with our 2-generation pedigree. We used ASReml-R v. 3.0 [62] to fit linear mixed effects models with (1) selected variables in the previous models of nymphal development as fixed effects and (2) pedigree (and individual identity for growth rate) as a random effect (see above the section ‘Pedigree and quantitative genetics design‘). For each trait and each sex separately, we estimated the genetic variance (*V*_*G*_), residual variance (*V*_*R*_) and broad-sense heritability (*H*^*2*^: *V*_*G*_*/(V*_*G*_*+V*_*R*_*)*), along with associated standard errors (*SE(V*_*G*_*)*, *SE(V*_*P*_*)* and *SE(H*^*2*^*)*).

Since molt strategy is a binary trait, we used the threshold model described in [63] to estimate its heritability. Briefly, molt strategy is considered to depend on an underlying trait, called liability, which has a continuous distribution. If liability reaches a certain threshold value, the focus individual is considered to undergo an extra-molt. Since liability is normally distributed, the heritability estimate for molt strategy was obtained by using the standard quantitative genetics method described above and then applying a correction [64,65]. Unfortunately, this method cannot be used to estimate component variances. While it would have been possible to use a Bayesian animal model as an alternative method, our limited sample size would have implied a strong bias in estimation [66].

Since genetic variance is not necessarily independent of environmental variance, variance scaling imposed by heritability calculations can be unreliable to compare genetic parameters between traits [20]. Therefore, we derived the genetic and residual coefficients of variation (*CV*_*G*_ and *CV*_*R*_, respectively), which are less affected by (potentially uncontrolled) residual variance [20], *as CV*_*G*_ *= 100*.*√V*_*G*_ */ μ* and *CV*_*R*_ *= 100*.*√V*_*R*_ */ μ*, with *μ* the phenotypic mean of the trait [22]. We also computed evolvability estimates under stabilizing selection based on mean scaling, which represents the percent change in a trait under unit strength of selection, as *e*_*μ*_ *= V*_*G*_*/μ*^*2*^ [22].

All analyses were performed with R v. 3.2.3 (Development Core Team, 2011).

## Results

### Survival analysis

**Fig 1:**
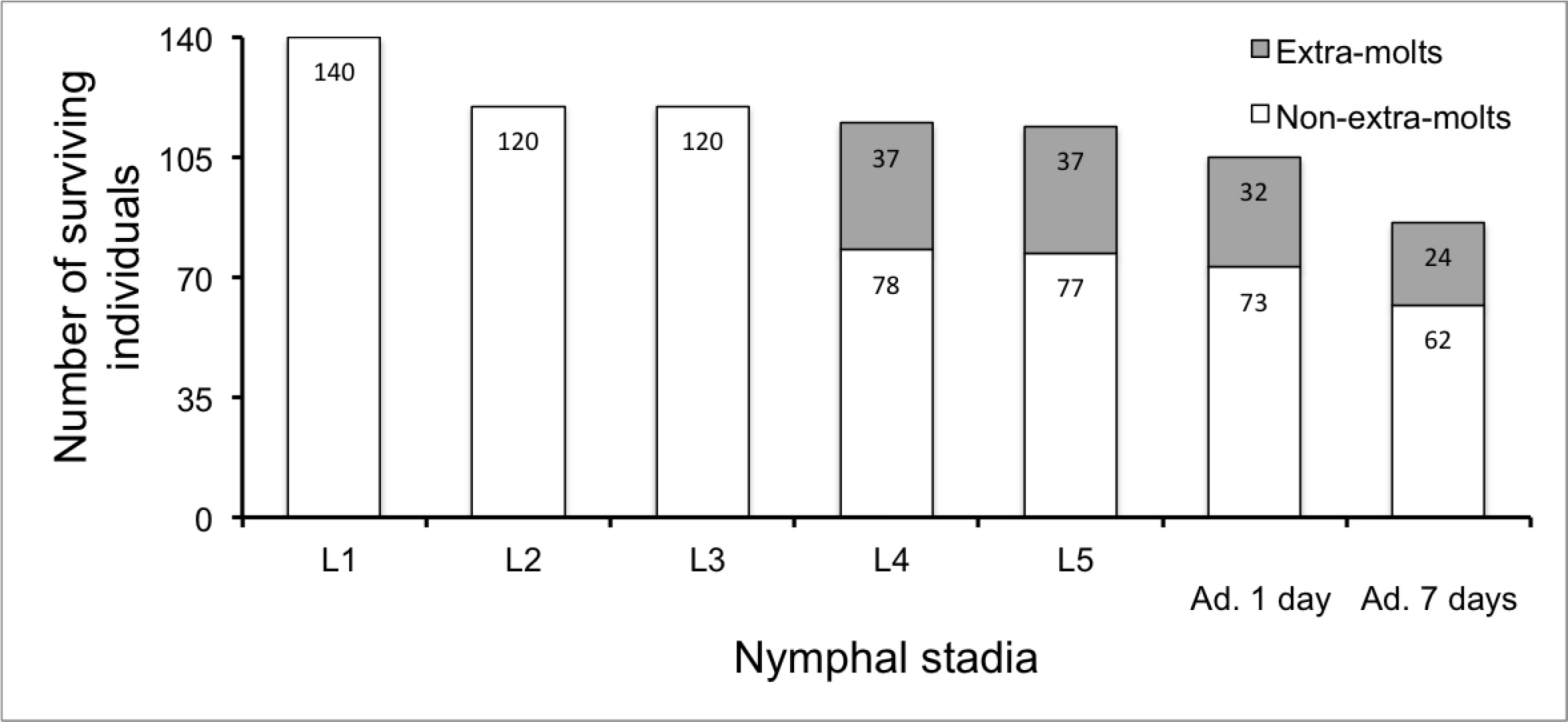
Number of surviving individuals by nymphal stadium and molt strategy. Grey parts represent the fraction of extra molting individuals beyond the L3 stadium. Sample sizes are indicated within bars. NB: we monitored the nymphal growth of the 105 individuals that reached adulthood plus 5 individuals that reached the L5 instar but failed to molt as adults (among 114 L5).

Fig 1 shows survival data for each life stage transition from 1^st^ nymphal instar to immature adults. GLMs did not reveal any differences in stage-by-stage survival probabilities between sexes and between molt strategies (*P* > 0.05). However, the probability of survival after the stage at which extra molt occurs (i.e. from the fourth nymphal instar to the seventh day of adult life) was lower for extra molting individuals (0.75 *versus* 0.88), though only approaching the standard level of significance (*F* = -1.789, *p* = 0.073), which can partly be ascribed to our limited sample size.

### Nymphal development

**Fig 2:**
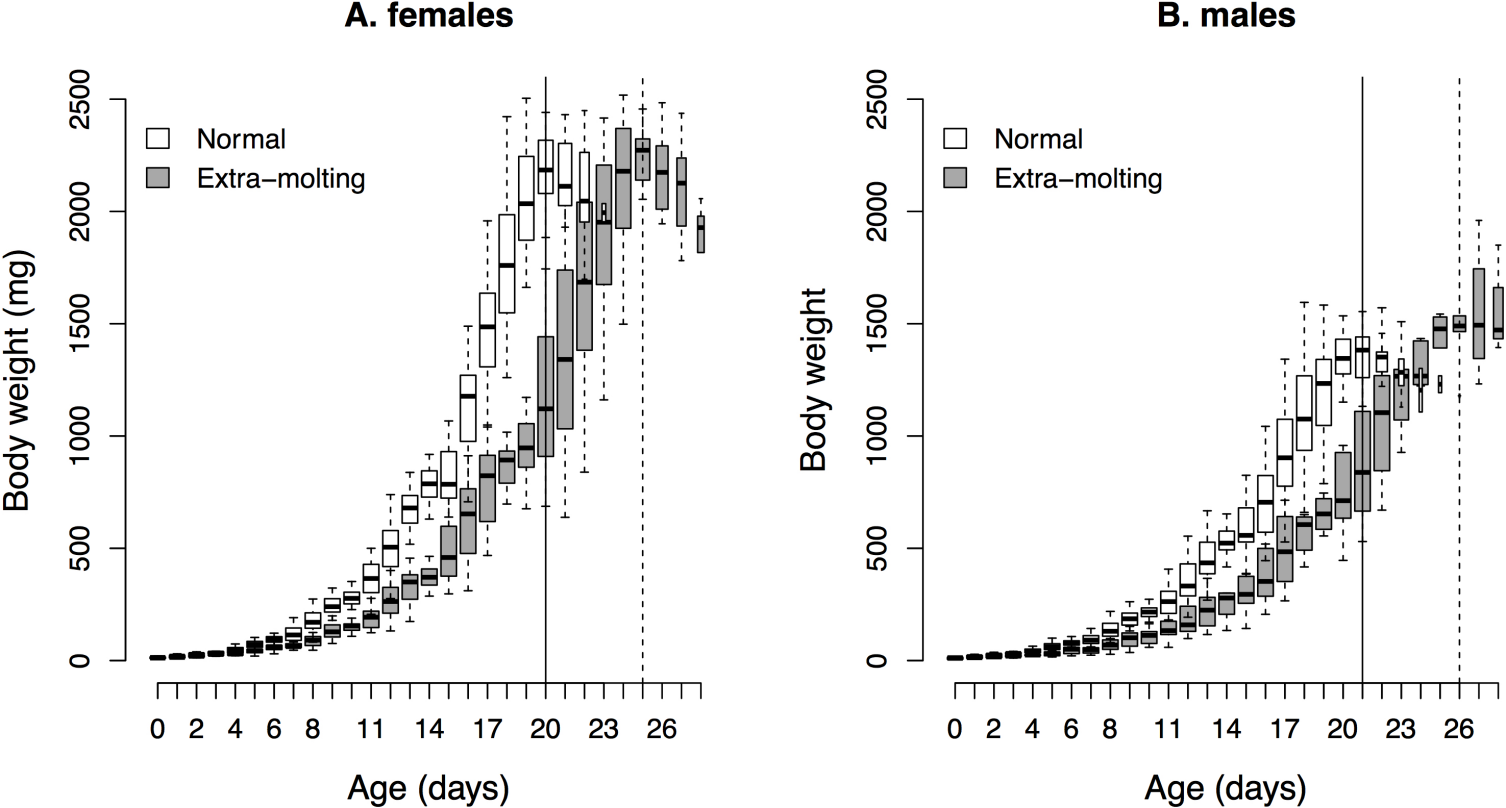
Nymphal growth in *S*. *gregaria*, displayed as body weight against age. A: for females only, B: for males only. For each box, black horizontal lines represent medians and notches represent its 95% confidence interval. Box width is proportional to the number of individuals in each group. Solid and dotted vertical lines represent the ages at which the maximal nymphal weight is reached in normal and extra-molting individuals respectively.

Table 1 shows the number of individuals and average hatch weight for each molt strategy and sex. We included in the analysis of nymphal growth the 110 individuals that emerged as adults and for which maximal nymphal weight could be estimated. Statistical tests and predicted estimates for the four developmental traits are provided for the model with the lowest AIC score only (Table 2) and for all models, including those displaying a ΔAIC < 2 (S1 Table). Fig 2 depicts the integrative growth response based on actual data (rather than models outputs).

**Table 1:** Distribution of the 110 surviving individuals by sex and by development strategy. Average hatch weight (in mg) for each class are indicated between brackets (mean ± sd).

Factors	Females	Males	Total
**Extra molting**	27 (10.37 ± 2.11)	7 (8.86 ± 2.53)	34 (10.06 ± 2.49)
**Non-extra molting**	31 (13.19 ± 2.75)	45 (12.38 ± 3.13)	76 (12.71 ± 2.99)
**Total**	58 (11.879 ± 2.83)	52 (11.804 ± 3.37)	110 (11.891 ± 3.09)

**Table 2:** Factors influencing nymphal development in *S*. *gregaria*. For each trait, selected variables came from linear models displaying the lowest AIC score, among all possible models including a null model and a full model (i.e., containing all variables as well as every simple interaction between pairs of variables; see Methods). Note that for extra molting and for growth rate we used GLMs with logit link function and linear mixed model, respectively. We report estimates, standard deviations, *t* values (except for extra molting; #: *z* values) and *P*-values associated with each variable (and intercepts). For growth rate, L2, L3, L4 and L5 refer to 2nd to 5th nymphal instars. L3b refers to the added nymphal instar for individuals undergoing extra molting.

Trait	Selected variables	Estimate	Std. error	t	P-value	Sig.
**Extra molting #**	(Intercept)	4.506	1.142	3.947	7.910E-05	***
	hatch weight (male)	-0.390	0.094	-4.153	3.280E-05	***
	sex (male)	-2.104	0.563	-3.739	1.850E-04	***
**Maximal nymphal weight**	(Intercept)	1952.291	118.119	16.528	< 2E-16	***
	hatch weight (male)	20.083	8.864	2.266	2.569E-02	*
	sex (male)	-533.119	134.029	-3.978	1.340E-04	***
	extra molting (yes)	157.669	42.881	3.677	3.870E-04	***
	hatch weight: sex (male)	-22.239	10.571	-2.104	3.799E-02	*
**Development time**	(Intercept)	21.828	0.620	35.205	< 2E-16	***
	hatch weight (male)	-0.041	0.044	-0.935	3.520E-01	NS
	sex (male)	0.281	0.284	0.989	3.251E-01	NS
	extra molting (yes)	1.933	1.133	1.706	9.120E-02	NS
	hatch weight: extra molting (yes)	0.253	0.099	2.561	1.200E-02	*
	sex (male): extra molting (yes)	0.932	0.588	1.584	1.165E-01	NS
**Growth rate**	(Intercept)	0.529	0.027	19.588	< 2E-16	***
	L2	-0.125	0.033	-3.817	1.550E-04	***
	L3	-0.131	0.033	-4.013	7.060E-05	***
	L3b	-0.220	0.062	-3.571	3.930E-04	***
	L4	-0.224	0.033	-6.853	2.480E-11	***
	L5	-0.314	0.033	-9.601	< 2E-16	***
	extra molting (yes)	0.019	0.030	0.635	5.265E-01	NS
	hatch weight (male)	-0.013	0.002	-6.324	5.980E-10	***
	sex (male)	-0.030	0.007	-4.269	4.400E-05	***
	hatch weight: extra molting (yes)	-0.006	0.003	-2.030	4.477E-02	*
	L2: hatch weight	0.011	0.003	4.221	2.970E-05	***
	L3: hatch weight	0.010	0.003	3.854	1.340E-04	***
	L3b: hatch weight	0.016	0.006	2.714	6.910E-03	**
	L4: hatch weight	0.013	0.003	5.027	7.310E-07	***
	L5: hatch weight	0.015	0.003	5.482	7.150E-08	***

*** < 0.001 < ** < 0.01 < * < 0.05 <. < 0.1

**Fig 3:**
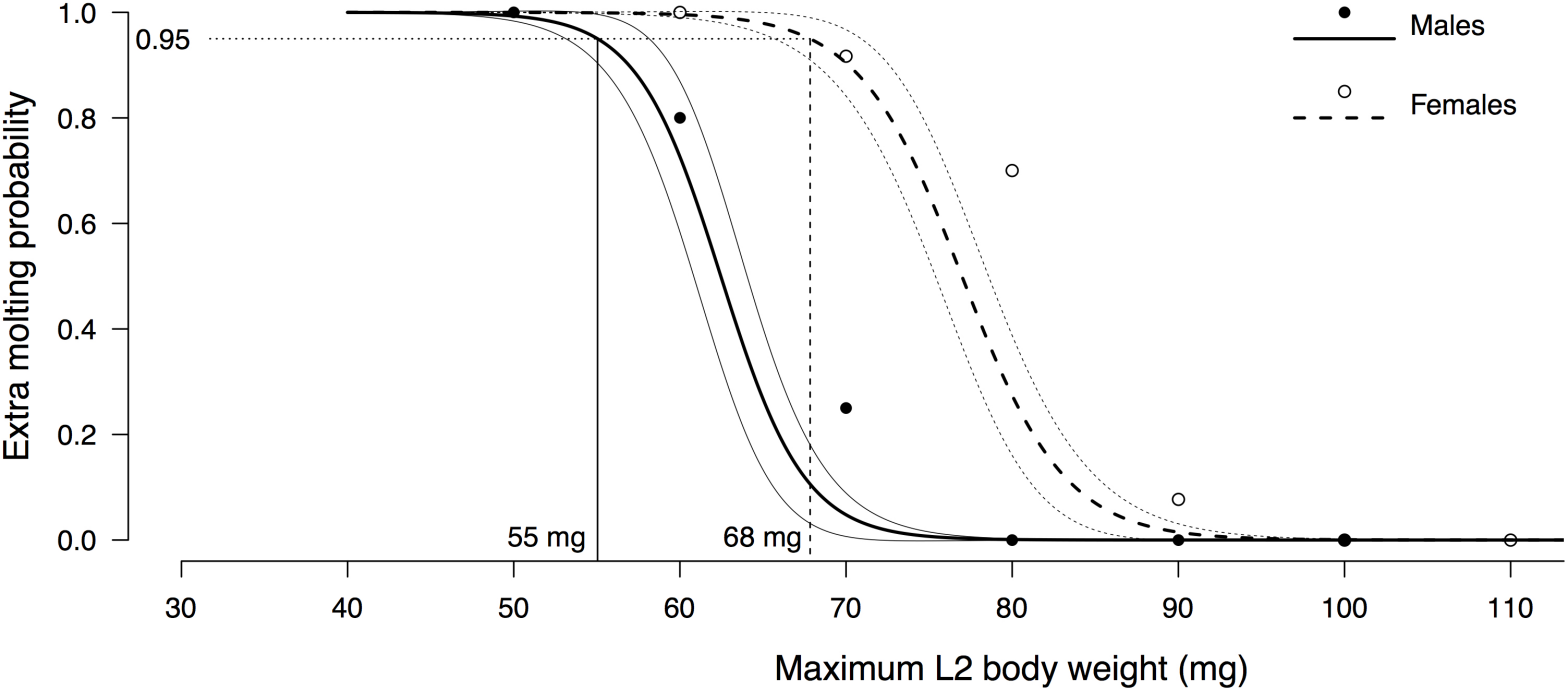
Extra molting probability for each sex as a function of L2 maximum body weight. Circles (filled for males and open for females) represent the proportion of extra molting observed in groups of individuals every 10mg of maximum L2 body weight. Curves (continuous for males and dashed for females) represent the fit (and associated standard error) of the following GLM logistic regression model: (extra molting)~(maximum L2 body weight):(sex) (backwardly-selected by AIC). The horizontal dotted line shows the 95% probability above which individuals undergo an extra molt. Vertical lines show the corresponding body weight thresholds for each sex, i.e. the 2nd-instar body weight under which the predicted extra-molting probability exceeded 95%: 55mg for males and 68mg for females.

The average probability for an individual to undergo an extra molt was 0.309. As expected, GLM analyses show that large hatch weight (*estimate ± se* = -0.39 ± 0.094, *z* = -4.153, *P* = 0.033E-3) and male sex (*estimate ± se* = -2.103 ± 0.563, *z* = -3.739, *P* = 0.185E-3) had a significant negative effect on the probability of extra molting. Indeed, 13.5% of the males performed an extra-molt compared with 46.5% of the females (Table 1). While average hatch weight was not different in the two sexes (one-way anova: *F*_*1*,*108*_ = 0.002, *P* = 0.967), it was higher in individuals that developed directly compared with individuals undergoing extra molting (12.7mg *vs*. 10.1mg, respectively; Table 2). Fig 3 shows that the body weight threshold, i.e., the maximal 2^nd^-instar body weight under which the predicted extra molting probability exceeded 95%, was 55mg for males and 68mg for females.

Growth rate varied among instars but tended to decrease over the developmental period, with a mean estimate varying from 0.529 in the first instar to 0.215 in the last instar (Table 2). Growth rate was also significantly affected by all the other explanatory variables, as well as by the interactions between hatch weight and nymphal instar and between hatch weight and extra molting. Once nymphal instar was taken into account, sex had the strongest effect on growth rate, followed by extra molting and hatch weight (Table 2). Male larvae respectively grew on average 5.7% more slowly than female individuals (Table 2; Fig 2). Hatch weight had a significant negative effect on growth rate during the first nymphal instar only, meaning that smaller offspring, independently of their sex or molt strategy, displayed a higher growth rate (Pearson’s correlation: -0.4, *P* <0.0001; Table 2). Note that two alternative models displayed a ΔAIC < 2 (ΔAIC = 1.81 and 1.94, respectively; Table 2 and S1 Table), although none of the added terms (mostly interactions) turned out to be significant and the estimates for other variables were not significantly impacted by their inclusion.

Development time was significantly affected by the interaction between hatch weight and molt strategy only (Table 2). Surprisingly, our selected model also included four terms with a non-significant *P*-value (sex, *P* = 0.32; extra molting, *P* = 0.09; hatch weight, *P* = 0.35 and the interaction between sex and extra molting, *P* = 0.12). Three alternative models displayed ΔAIC < 2 (ΔAIC = 0.22, 0.63 and 1.8, respectively; S1 Table), with two of them showing unchanged estimates and non-significant new terms. However, one of them (ΔAIC = 0.63) did not include the interaction between sex and extra molting, which brought out two significant effect that were otherwise hidden: sex (*estimate ± se* = 0.499±0.251, *P* = 0.049) and extra molting (*estimate ± se* = 2.6±1.06, *P* = 0.016), supporting the idea of a significant effect of those explanatory variables on development time. Indeed, our data show that extra molting insects had the longest development time, as the imaginal molt was reached in 26.2 days on average after hatching, compared with 21.5 days for insects that developed in 5 nymphal stages (Fig 2). Conversely, comparisons between sexes revealed minute differences, with a mean of 20 days for females and 20.5 days for males (Fig 2).

Maximal nymphal weight was strongly affected by sex (males weighed 37.5% less than females), extra molting (gain of 4% within females), hatch weight (weight increase of 1.14% per mg of hatch weight) and the interaction between hatch weight and sex, which revealed no effect of hatch weight in males (Table 2; see also Fig 2). For this trait, four alternative models displayed ΔAIC values < 2 (from 0.84 to 1.61), without impacting the estimates of explanatory variables present in the selected model and only adding non-significant terms (Table 2).

### Heritability and genetic correlations of growth traits

Table 3 shows genetic and residual variances (*V*_*G*_ and *V*_*R*_, respectively), heritabilities (*H*^*2*^), coefficients of variation (*CV*_*G*_ and *CV*_*R*_, respectively) and evolvability estimates (*e*_*μ*_) obtained from univariate animal models (except for the 'extra molting' binary trait, for which only heritability could be calculated). Note that genetic estimates based on models with ΔAIC < 2 did not vary significantly, for any of the analyzed trait (data not presented). Maximal nymphal weight was the only trait to show null genetic parameters, including heritability and evolvability, mostly due to a *CV*_*R*_ 4.4 times higher than *CV*_*G*_. Although all evolvability estimates were almost null (due to large sample means relative to variances captured by our design), growth rate, development time and molt strategy all showed substantial heritability estimates. Growth rate exhibited a low heritability estimate of 0.13 and low standard errors of 0.05, with a P-value of 0.006 and no difference between sexes. On the contrary, development time showed a higher heritability value of 0.39, though associated with a high standard error of 0.33 and a *P*-value just above the standard significance level (*P* = 0.053). Heritability of extra molting turned out to be quite high at 0.67, with a fairly low standard error of 0.37 and a *P*-value of 0.016. Interestingly, extra molting and development time displayed high heritabilities values (>0.5) only for females (*P* = 0.035 and *P* = 0.053, respectively) and a null heritability for males (*P* = 0.519 and *P* = 1, respectively). Finally, growth rate showed significant overall heritability, although being non-significant for both sexes when considered separately (Table 3).

**Table 3:** Genetic parameters of each trait of nymphal development in *S*. *gregaria*.

Traits	Group	*μ*	*V*_*G*_	*V*_*R*_	*H*^*2*^	*CV*_*G*_	*CV*_*R*_	*e*_*μ*_	*P-val*.
**Development time**	All	22.90	6.22E-01	9.55E-01	0.39 ± 0.33	3.44	4.27	1.18E-03	0.053
	F	23.44	6.02E-01	5.82E-01	0.51 ± 0.42	3.31	3.25	1.09E-03	0.053
	M	22.34	3.12E-07	1.64	<10E-3 ± <10E-3	2.5E-03	5.73	6.25E-10	1
**Maximal nymphal weight**	All	1848.58	1.31E+03	2.59E+04	0.05 ± 0.13	1.96	8.71	3.83E-04	0.683
	F	2264.90	3.92E-03	3.97E+04	<10E-3 ± <10E-3	2.76E-03	8.80	7.64E-10	1
	M	1415.60	2.29E-02	1.43E+04	<10E-3 ± <10E-3	0.01	8.45	1.14E-08	1
**Extra molting**	All	0.32	_	_	0.67 ± 0.37	_	_	_	0.016
	F	0.47	_	_	>1 ± 0.57	_	_	_	0.035
	M	0.15	_	_	0.18 ± 0.31	_	_	_	0.519
**Growth rate**	All	0.30	5.68E-04	3.95E-03	0.13 ± 0.05	7.94	20.95	6.31E-03	0.008
	F	0.31	5.62E-04	4.21E-03	0.14 ± 0.06	7.65	20.93	5.85E-03	0.085
	M	0.30	3.61E-04	3.63E-03	0.09 ± 0.12	6.33	20.08	4.01E-03	0.49

*μ*: phenotypic mean; *V*_*G*_: genetic variance; *V*_*R*_: residual variance; *H*^*2*^: broad-sense heritability (± standard error) (computed as *V*_*G*_*/(V*_*G*_*+V*_*R*_*)*); *CV*_*G*_: genetic coefficient of variation (*100*.*√V*_*G*_ */ μ*); *CV*_*R*_: residual coefficient of variation (*100*.*√V*_*R*_ */ μ*); *e*_*μ*_: evolvability (*V*_*G*_ */ μ*^*2*^). /: estimate not available.

## Discussion

In this study, we observed a high propensity to express an extra nymphal instar (at least in females: 46.5%) within a solitarious population of *S*. *gregaria*, reared under optimal laboratory conditions for three generations since field sampling. There are few available data from natural solitarious populations of desert locusts for comparison. However, in the field (non-optimal) conditions of central Sahara, 89% of females and 46% of males were reported to have undergone an extra molt [38]. In winter populations of the Southern African *S*. *g*. *flaviventris*, a desert locust subspecies with a lower propensity to gregarise, 50% of females and 26% of males were found to have developed with extra molt (i.e., showing eyes with 7 stripes, Marie-Pierre Chapuis and Antoine Foucart, pers. com; see also [67]).

### Phenotypic adjustment of instar number in *S*. *gregaria*

As in many species [10,23,24], extra molting in *S*. *gregaria* has long been viewed as a catch-up strategy [28,33,39,60]. We confirm here that undergoing an extra molt allows small-size desert locust hatchlings to compensate for low body weight by adjusting their developmental pathway. First, small hatch weight was associated with a significantly higher probability to undergo an extra molt. Second, lower body weight in six-instar hatchlings did not convert into lower maximal nymphal weight, and extra molting locusts became even significantly heavier than non-extra molting ones (i.e. 95mg or 4.3% for males and 198.1mg or 14.3% for females on average). A full compensation pattern in which the extra molt allows individuals of small initial body size to reach greater adult body size than individuals developing normally has already been reported in moths (e.g. [15]). Since in hemimetabolous insects nymphal molts are caused by ecdysones in the presence of juvenile hormones (JHs; [31]), differences in timing and composition of the hormones are likely to underlie the alternative development trajectories in the desert locust, although it still remains to be ascertained.

This catch-up growth strategy was complemented by hatchlings of smaller sizes increasing their growth rate during the first instar (compensatory growth). Such a mechanism of growth rate adjustment has already been observed in the red locust, *Nomadacris septemfasciata* [28,32], suggesting that it could be common to several locust species. Here however, this adjustment mechanism was less effective than extra molting, allowing a weight increase of 0.15 to 0.2% only of the maximal nymphal weight during the first-instar nymphal lifetime. As expected, the extra-molt strategy represents a developmental compromise with other growth parameters: at 34°C, extra-molting nymphs took ~22% longer to develop (i.e. five more days), thus decreasing their overall growth rate by an average of ~8%. Interestingly, it may also imply a fitness trade-off between strategies, as we found a 15% lower probability of survival until adulthood (between the 4^th^ nymphal instar and the first week of adult life in our analyses) for individuals that went through an additional nymphal molt. Since our estimates were not significant (which may partly be due to our limited sample size), we cannot conclude firmly on this point. Note however that a negative correlation between extra molting and survival was already reported in the desert locust [68].

While hatch weight is the same in males and females, maximal nymphal weight is 850mg lower in males (1415mg and 2265mg for males and females respectively), implying a 1.6 female-biased sexual size dimorphism (SSD). This strongly suggests that the body weight threshold under which young nymphs (2^nd^ instar) add one extra instar to their development is different in the two sexes. According to our data, the 95% probability estimate of this critical body weight was ~20% higher in females than in males (68mg and 55mg respectively; Fig 3). This translated into a strong sexual dimorphism in extra molting, with females producing an extra-molt 3 times more often than males (nearly half the females underwent an extra-molt), in accordance with the observed female-biased SSD. A female-biased extra molting probability associated with a female-biased SSD are in agreement with previous studies in *S*. *gregaria* [60] and common in many other insect species [6,10]. Interestingly, the weight at first day of the third instar was 1.5 greater for non-extra molting females than for extra molting ones (i.e., 77.24 mg vs. 115.91 mg, respectively), which was comparable to what Maeno & Tanaka ([60]) measured in their study (i.e., 79.2 mg vs. 110.3 mg).

### Selection on nymphal growth in *S*. *gregaria*

Our quantitative genetics design allowed, for the first time in locusts, to account for the genetic contribution to phenotypic variance of four key nymphal developmental traits (instar number, growth rate, maximal weight and development time), thus shedding light on their potential to respond to selection. However, our estimates of genetic parameters should be evaluated with caution. First, our dataset was limited to 110 individuals and 14 full-sibs families, potentially decreasing our power to detect significant genetic variation. This potential bias seems to have little impacted our analyses since most of our heritability estimates were significant, though it probably contributes to the relatively large confidence intervals of these estimates. Second, despite the fact that we used pedigree data, our quantitative genetics design was based on phenotyping mainly full-sibs, thus not allowing us to control for maternal effects. It could be considered, for example, that maternal ecdysteroids responsible for the molts during the embryonic development may also have delayed effects in the young nymph [34,69,70]. It is however important to note that in our analyses maternal effects related to hatchling size were directly accounted for by adding hatch size as a fixed factor in our quantitative genetics models.

The high prevalence of extra-molt events emphasizes the fact that body weight at adult emergence might be the target of nymphal growth in *S*. *gregaria*. Here, approximated by the maximal nymphal weight, this trait showed negligible heritability and evolvability estimates in both sexes, due to a 4.4 times lower genetic *vs*. residual mean-standardized variance (Table 3). This is in agreement with most studies in insects, though high heritability estimates are sometimes found with no clear explanation (e.g. [71]). The body weight threshold for adult emergence therefore seems to be genetically canalized, leaving no genetic variance for selection to act on. Altogether, these quantitative genetics results suggest that body weight is under strong selection in the desert locust [20,21,72]. In invertebrates, a gain in body weight can usually be converted into greater offspring production [1] and it has been shown that extra molting females of the desert locust did produce a larger number of offspring (at the cost of their size and quality; [37]). In addition, a gain in body weight can offer greater resistance to starvation [73], which is interesting in the case of the desert locust, since this species is adapted to the high climatic stochasticity of desert habitats. It seems plausible that the energy costs of homeostasis in a low quality environment and of long-distance migration in search for more vegetated areas suitable for reproduction represent a strong selective component favoring large adult body weight in *S*. *gregaria*.

In contrast, estimates of heritability for growth rate, development time and extra molting were fairly high (0.13, 0.39 and 0.67, respectively; Table 3), suggesting that ongoing selection on nymphal growth can still be directed toward those traits. On the one hand, the lowest estimate of overall heritability among those three traits was found for growth rate, likely due to strong physiological constraints within the successive instars, each only lasting a few days [9]. These results are in agreement with a previous study focused on the field cricket *Gryllus pennsylvanicus*, in which growth rate and development time both exhibited high heritability and were genetically correlated [74]. Unfortunately, we cannot tell whether these traits could constrain each other’s evolution in the desert locust, since we could not calculate genetic correlations due to a lack of statistical power.

On the other hand, extra molting showed by far the highest genetic contribution to the observed phenotypic variance (*H*^*2*^ = 0.67). These results suggest that in *S*. *gregaria* extra molting might be more prone to respond to selection (and thus to evolve rapidly) than the other growth traits we studied, although this would need to be formally investigated by calculating selection gradients in natural populations. Yet, our experimental and statistical design did not fully control for maternal effects and we cannot exclude that a portion of the genetic variance captured in our analyses was in fact non-genetic. Nevertheless, the evolutionary potential of this developmental trait was indirectly suggested by the findings of previous studies in insects (e.g., [14,15,28,75]). In the desert locust, repeated selection of 6-instar nymphs only as parents for the next five generations increased the frequency of extra molting females from 20% to 94% [37], suggesting that this trait is heritable, which we confirm in the present study.

Extra-molt exhibited not only the greatest genetic variance, but also an extreme discrepancy between sexes, with only the females showing a significant heritability close to 1. This result is consistent with the findings from [37]’s artificial selection experiment on extra-molt, which showed a response to selection in the first generation that fits, in their case, the realized heritability of 8% for males and 50% for females. Here, genetic variance and sexual dimorphism of extra molting were both strongly biased toward females, which suggests that this trait is under strong positive selection in this sex. In insects, because of the inherent cost to sexual reproduction, females are usually under stronger selection than males to reach larger adult body weights [1,2,5]. Our results suggest that selective pressures on nymphal development diverge between sexes in the desert locust, generating a sexual dimorphism in growth trajectories [16] with, in particular, extra molting allowing females to reach a higher body weight than males at adult emergence [6].

In addition, such female genetic contribution and genetic conflict with males may explain why we observed a large number of extra instars in optimal conditions of temperature and food resources, although these conditions predict a prevalence of normal growth (e.g. [14]). Unfortunately, due to limited sample sizes, we could not verify whether this trait showed a strong positive intersexual genetic correlation to further support this hypothesis. It is worth noting that sexual, female-biased, antagonistic selection on nymphal growth was also apparent in the sex-specific differences in development time heritability. To thoroughly resolve whether sexual conflict is ongoing on extra molting in the desert locust, measures of fitness for each sex and each developmental strategy are required along with the genetic covariance between the trait values and fitness [17,76].

Anyhow, the substantial within-population genetic variation observed for the number of nymphal instars in optimal conditions for development probably indicates that this trait has not yet been fixed at its optimum by natural selection. In addition to sexual conflict, antagonistic selection between heterogeneous environments may explain the maintenance of high level of genetic variance for this trait [77]. First, in populations of a close species, the red locust, the rate of extra molting in the field was shown to decrease gradually during the dry season, which might be a sign of a differential selection on growth traits during the favorable and the unfavorable seasons in this species [28,41]. Second, determinants of natural selection might differ between low-density (solitarious) and high-density (gregarious) populations [32,33,60], since extra molting is almost absent in gregarious populations of *S*. *gregaria*. The fitness cost of a longer exposition to predation, disease [78] and cannibalism [79,80] in high-density populations might counter-select extra-molting individuals. The production of bigger hatchlings [32,81] and the existence of a much reduced adult SSD [33,34] in gregarious populations might lower the intensity of selection for the catch-up strategy. Finally, genetic correlations between growth and phase traits may prevent the fixation of the number of nymphal instars. Phenotypic correlations between density-dependent instar number and adult morphology were already demonstrated in the desert locust, with, for instance, shorter-winged 6-instar adults [82]. Unfortunately, measuring with precision the genetic component of growth traits of offspring reared in (gregarious) high density conditions is very difficult because of the lack of reliable methods for marking individual Orthopteran larvae durably throughout their development and successive molts [83]. This leaves open the question of the potential adaptiveness of extra molting in natural populations and of its role in the evolution and maintenance of phase polyphenism in locusts.

## Supporting Information

S1 Table Factors influencing nymphal development in *S*. *gregaria*, for all models, including those displaying a ΔAIC < 2. For each trait, selected variables came from linear models displaying a ΔAIC < 2, among all possible models including a null model and a full model (i.e., containing all variables as well as every simple interaction between pairs of variables; see Methods). Note that for extra molting and for growth rate we used GLMs with logit link function and linear mixed model, respectively. We report estimates, standard deviations, *t* values (except for extra molting; #: *z* values) and *P*-values associated with each variable (and intercepts). For growth rate, L2, L3, L4 and L5 refer to 2nd to 5th nymphal instars. L3b refers to the added nymphal instar for individuals undergoing extra molting. Gray areas emphasize the outcome of models displaying the lowest AIC values. *** < 0.001 < ** < 0.01 < * < 0.05 <. < 0.1
(DOCX)

## References

[1] Andersson M. Sexual selection Krebs J, Clutton-Brock T, editors. Princeton: Princeton University Press; 1994.

[2] Honěk A. Intraspecific variation in body size and fecundity in insects: a general relationship. Oikos. Wiley on behalf of Nordic Society Oikos; 1993;66: 483–492.

[3] Møller AP, Jennions MD. How important are direct fitness benefits of sexual selection. Naturwissenschaften. 2001;88: 401–415. 11729807 10.1007/s001140100255

[4] Rodríguez-Muñoz R, Bretman A, Slate J, Walling CA, Tregenza T. Natural and sexual selection in a wild insect population. Science. 2010;328: 1269–1272. 10.1126/science.1188102 20522773

[5] Stearns SC. The evolution of life histories Oxford University Press; 1992 pp. 249–249.

[6] Esperk T, Tammaru T, Nylin S, Teder T. Achieving high sexual size dimorphism in insects: females add instars. Ecological entomology. Wiley Online Library; 2007;32: 243–256.

[7] Whitman DW, Agrawal AA. What is phenotypic plasticity and why is it important In: Whitman DW, Ananthakrishnan TN, editors. Phenotypic plasticity of insects: Mechanisms and consequences. Science Publishers: Enfield, NH, USA; 2009 pp. 1–63.

[8] Gotthard K. Growth strategies of ectothermic animals in temperate environments In: Atkinson D, Thorndyke MC, editors. Animal Developmental Ecology. Oxford: Oxford: BIOS Scientific, 2001; 2001. pp. 1–18.

[9] Greenlee KJ, Harrison JF. Respiratory changes throughout ontogeny in the tobacco hornworm caterpillar,Manduca sexta. J Exp Biol. England; 2005;208: 1385–1392. 10.1242/jeb.01521 15781898

[10] Esperk T, Tammaru T, Nylin S. Intraspecific variability in number of larval instars in insects. J Econ Entomol. 2007;100: 627–645. 17598520 10.1603/0022-0493(2007)100[627:ivinol]2.0.co;2

[11] Stillwell RC, Blanckenhorn WU, Teder T, Davidowitz G, Fox CW. Sex differences in phenotypic plasticity affect variation in sexual size dimorphism in insects: from physiology to evolution. Annu Rev Entomol. United States; 2010;55: 227–245. 10.1146/annurev-ento-112408-085500 19728836 PMC4760685

[12] Nijhout HF. A threshold size for metamorphosis in the tobacco hornworm,Manduca sexta(L.). Biological Bulletin. 1975;149: 214–214. 1138977 10.2307/1540491

[13] Auer SK, Arendt JD, Chandramouli R, Reznick DN. Juvenile compensatory growth has negative consequences for reproduction in Trinidadian guppies (Poecilia reticulata). Ecol Lett. 2010;13: 998–1007. 10.1111/j.1461-0248.2010.01491.x 20545728

[14] Kingsolver JG. Variation in growth and instar number in field and laboratoryManduca sexta. Proc Biol Sci. England; 2007 10.1098/rspb.2006.0036 PMC2141666 17251106

[15] Saastamoinen M, Ikonen S, Wong SC, Lehtonen R, Hanski I. Plastic larval development in a butterfly has complex environmental and genetic causes and consequences for population dynamics. J Anim Ecol. 2013;82: 529–539. 10.1111/1365-2656.12034 23347450

[16] Blanckenhorn WU. Behavioral causes and consequences of sexual size dimorphism. Ethology. Wiley Online Library; 2005;111: 977–1016. 10.1111/j.1439-0310.2005.01147.x

[17] Cox RM, Calsbeek R. Sexually antagonistic selection, sexual dimorphism, and the resolution of intralocus sexual conflict. Am Nat. 2009;173: 176–187. 10.1086/595841 19138156

[18] Wyman MJ, Rowe L. Male bias in distributions of additive genetic, residual, and phenotypic variances of shared traits. Am Nat. 2014;184: 326–337. 10.1086/677310 25141142

[19] Reinhold K, Engqvist L. The variability is in the sex chromosomes. Evol. 2013;67: 3662–3668. 10.1111/evo.12224 24299417

[20] Hansen TF, Pélabon C, Houle D. Heritability is not evolvability. Evol Biol. Springer US; 2011;38: 258–277.

[21] Debat V, David P. Mapping phenotypes: canalization, plasticity and developmental stability. Trends in Ecology & Evolution. Elsevier Ltd; 2001;16: 555–561. 10.1016/S0169-5347(01)02266-2

[22] Houle D. Comparing evolvability and variability of quantitative traits. Genetics. Genetics Society of America; 1992;130: 195–204. 1732160 10.1093/genetics/130.1.195 PMC1204793

[23] Nijhout HF. A threshold size for metamorphosis in the tobacco hornworm,Manduca sexta(L.). Biological Bulletin. 1975;149: 214–214. 1138977 10.2307/1540491

[24] Telfer MG, Hassall M. Ecotypic differentiation in the grasshopper Chorthippus brunneus: life history varies in relation to climate. Oecologia. Springer-Verlag; 1999;121: 245–254. 10.1007/s004420050926 28308564

[25] Hassall M, Grayson FWL. The occurrence of an additional instar in the development of Chorthippus brunneus (Orthoptera: Gomphocerinae). Journal of Natural History. 1987;21: 329–337. 10.1080/00222938700771051

[26] Mathur CB. An extra hopper stage in the Desert Locust (Schistocerca gregariaForsk.). 1938. p. 177.

[27] Mukerji S, Batra RN. A note on the post-embryonic development of eye-stripes and their correlation with the number of larval instars and the antennal segments in the life-cycle ofSchistocerca gregariaForsk Brussels, Minist. Colon; 1938 pp. 410–415.

[28] Albrecht FO. La densitédes populations et la croissance chezSchistocerca gregaria(Forsk.) et Nomadacris septemfasciata (Serv.); la mue d'ajustement. Journal d'agriculture tropicale et de botanique appliquée. Muséum National d'Histoire Naturelle; 1955;2: 109–192. 10.3406/jatba.1955.2212

[29] Clynen E, Huybrechts J, Verleyen P, De Loof A, Schoofs L. Annotation of novel neuropeptide precursors in the migratory locust based on transcript screening of a public EST database and mass spectrometry. BMC Genomics. 2006;7: 201 10.1186/1471-2164-7-201 16899111 PMC1574313

[30] Závodská R, Sauman I, Sehnal F. Distribution of PER protein, pigment-dispersing hormone, prothoracicotropic hormone, and eclosion hormone in the cephalic nervous system of insects. J Biol Rhythms. 2003. 10.1177/0748730403251711 12693866

[31] Riddiford LM. Juvenile hormone action: a 2007 perspective. J Insect Physiol. 2008;54: 895–901. 10.1016/j.jinsphys.2008.01.014 18355835

[32] Hunter-Jones P. Laboratory studies on the inheritance of phase characters in locusts. Anti-Locust Bull. London. London; 1958.

[33] Uvarov BP. Grasshoppers and locusts: a handbook of general acridology. Volume 1. Anatomy, physiology, development, phase polymorphism, introduction to taxonomy Published for the Anti-Locust Research Centre at the University Press; 1966.

[34] Pener MP, Simpson SJ. Locust phase polyphenism: an update. Advances in Insect Physiology. Elsevier; 2009 pp. 1–272. 10.1016/S0065-2806(08)36001-9

[35] Simpson SJ, Sword GA. Locusts. Curr Biol. England; 2008;18: R364–6. 10.1016/j.cub.2008.02.029 18460311

[36] Song H. Density-dependent phase polyphenism in nonmodel locusts: a minireview. Psyche: A Journal of Entomology. 2011;2011: 1–16. 10.1155/2011/741769

[37] Injeyan HS, Tobe SS. Phase polymorphism inSchistocerca gregaria: reproductive parameters. Journal of Insect Physiology. Elsevier Ltd Elsevier; 1981;27: 97–102.

[38] Volkonsky M. Stries oculaires et âges larvaires chez les Acridiens. Arch Inst Pasteur Alger. 1938;4: 523–532.

[39] Maeno K, Tanaka S. Phase-specific developmental and reproductive strategies in the desert locust. Bull Entomol Res. England; 2008;98: 527–534. 10.1017/S0007485308006044 18590599

[40] Maeno K, Tanaka S. Patterns of nymphal development in the desert locust,Schistocerca gregaria, with special reference to phase-dependent growth and extra molting. Applied Entomology and Zoology. Japanese Society of Applied Entomology and Zoology; 2010;45: 513–519. 10.1303/aez.2010.513

[41] Albrecht FO, Blackith RE. Phase and moulting polymorphism in locusts. Evol. Blackwell Publishing Inc; 1957;11: 166–177.

[42] Joly P, Joly L. Résultats de greffes de Corpora allata chezLocusta migratoriaL. Ann Sci Nat, Zool. 1953;11: 331–345.

[43] Applebaum SW, Avisar E, Heifetz Y. Juvenile hormone and locust phase. Archives of Insect Biochemistry and Physiology. Wiley Online Library; 1997;35: 375–391. 10.1002/(SICI)1520-6327(1997)35:4<375::AID-ARCH3>3.0.CO;2-R

[44] Joly L. Résultats d'implantations systématiques de Corpora allata à de jeunes larves deLocusta migratoria. C R Soc Biol. 1954;148: 579–583. 13190760

[45] Joly P. Rôle joué par les Corpora allata dans la réalisation du polymorphisme de phase chezLocusta migratoriaL. 1962. pp. 77–88.

[46] Dirsh VM. Morphometrical studies on phases of the desert locust (Schistocerca gregariaForskal). Anti-Locust Bull. London; 1953;: 1–34.

[47] Nickerson B. Pigmentation of hoppers of the desert Locust (Schistocerca gregariaForskål) in relation to phase coloration. Anti-Locust Bull. London; 1956.

[48] Rungs CH. Une nouvelle représentation graphique de la grégariosité des populations du criquet pèlerin,Schistocerca gregariaForsk. 1954 pp. 130–132.

[49] Chapuis MP, Estoup A, Auge-Sabatier A, Foucart A, Lecoq M, Michalakis Y. Genetic variation for parental effects on the propensity to gregarise inLocusta migratoria. BMC Evol Biol. England; 2008;8: 37–37. 10.1186/1471-2148-8-37 18237445 PMC2276201

[50] Maeno K, Tanaka S. The trans-generational phase accumulation in the desert locust: morphometric changes and extra molting. J Insect Physiol. England; 2009;55: 1013–1020. 10.1016/j.jinsphys.2009.07.005 19631213

[51] Popov GB. Ecological studies on oviposition by swarms of the sesert locust (Schistocerca gregariaForskal) in eastern Africa. Anti-Locust Bull. London. London; 1958;: 70–70.

[52] Blondin L, Badisco L, Pagès C, Foucart A, Risterucci A-M, Bazelet CS, et al Characterization and comparison of microsatellite markers derived from genomic and expressed libraries for the desert locust. J Appl Entomol. 2013;137: 673–683. 10.1111/jen.12052

[53] Jones OR, Wang J. COLONY: a program for parentage and sibship inference from multilocus genotype data. Mol Ecol Resour. England; 2010;10: 551–555. 10.1111/j.1755-0998.2009.02787.x 21565056

[54] Albrecht FO. La densitédes populations et la croissance chezSchistocerca gregaria(Forsk.) et Nomadacris septemfasciata (Serv.); la mue d'ajustement. Journal d'agriculture tropicale et de botanique appliquée. Muséum National d'Histoire Naturelle; 1955;2: 109–192. 10.3406/jatba.1955.2212

[55] Rao YR, Gupta RL. Some notes on eye-stripes in Acrididae. Indian Journal of Agricultural Sciences. 1939;9: 727–729.

[56] Hughes TD. The imaginal ecdysis of the desert locust,Schistocerca gregaria. Physiological Entomology. Blackwell Publishing Ltd; 1980;5: 47–54. 10.1111/j.1365-3032.1980.tb00210.x

[57] Maeno K, Tanaka S. Artificial miniaturization causes eggs laid by crowd-reared (gregarious) desert locusts to produce green (solitarious) offspring in the desert locust,Schistocerca gregaria. J Insect Physiol. England; 2009;55: 849–854. 10.1016/j.jinsphys.2009.05.012 19505472

[58] Burnham KP, Anderson DR, Huyvaert KP. AIC model selection and multimodel inference in behavioral ecology: some background, observations, and comparisons. Behavioral Ecology and Sociobiology. Springer; 2011;65: 23–35. 10.1007/s00265-010-1029-6

[59] Burnham KP, Anderson DR. Multimodel Inference Understanding AIC and BIC in Model Selection. Sociological Methods & Research. SAGE Publications; 2004;33: 261–304. 10.1177/0049124104268644

[60] Maeno K, Tanaka S. Patterns of nymphal development in the desert locust,Schistocerca gregaria, with special reference to phase-dependent growth and extra molting. Applied Entomology and Zoology. Japanese Society of Applied Entomology and Zoology; 2010;45: 513–519. 10.1303/aez.2010.513

[61] Davidowitz G, D'Amico LJ, Nijhout HF. Critical weight in the development of insect body size. Evol Dev. 2003;5: 188–197. 10.1046/j.1525-142X.2003.03026.x 12622736

[62] Gilmour AR, Gogel BJ, Cullis BR, Welham SJ. ASReml user guide. Release 4.1 structural specification. VSN International Ltd 2014.

[63] Roff D. Estimation of heritability for threshold traits. Evolutionary quantitative genetics. Springer; 1997 pp. 493–493.

[64] Dempster ER, Lerner IM. Heritability of threshold characters. Genetics. Genetics Society of America Genetics Society of America; 1950;35: 212–212. 17247344 10.1093/genetics/35.2.212 PMC1209482

[65] Elston RC, Hill WG, Smith C. Query: estimating “heritability" of a dichotomous trait. Biometrics. JSTOR; 1977;: 231–236. 843575

[66] Morrissey MB, De Villemereuil P, Doligez B, Gimenez O. Bayesian approaches to the quantitative genetic analysis of natural populations In: Charmantier A, Garant D, Kruuk LEB, editors. Quantitative Genetics in the Wild. Oxford: Oxford University Press; 2014 pp. 228–253. 10.1093/acprof:oso/9780199674237.001.0001

[67] Chapuis M-P, Foucart A, Plantamp P, Blondin L, Leménager N, Benoit L, et al Genetic and morphological variation in non-polyphenic southern African populations of the desert locust. African entomology. 2016;24: In press.

[68] Maeno K, Tanaka S. The trans-generational phase accumulation in the desert locust: morphometric changes and extra molting. J Insect Physiol. England; 2009;55: 1013–1020. 10.1016/j.jinsphys.2009.07.005 19631213

[69] Hägele BF, Wang F-H, Sehnal F, Simpson SJ. Effects of crowding, isolation, and transfer from isolation to crowding on total ecdysteroid content of eggs inSchistocerca gregaria. J Insect Physiol. Elsevier; 2004;50: 621–628. 15234622 10.1016/j.jinsphys.2004.04.008

[70] Tawfik AI, Vedrova A, Sehnal FE. Ecdysteroids during ovarian development and embryogenesis in solitary and gregariousSchistocerca gregaria. Archives of Insect Biochemistry and Physiology. Wiley Online Library; 1999;41: 134–143. 10.1002/(SICI)1520-6327(1999)41:3<134::AID-ARCH4>3.0.CO;2-6/pdf 10398336

[71] Hammerschmidt K, Deines P, Wilson AJ, Rolff J. Quantitative genetics of immunity and life history under different photoperiods. Heredity. England; 2012;108: 569–576. 10.1038/hdy.2011.125 22187084 PMC3330682

[72] Price T, Schulter D. On the low heritability of life-history traits. Evol. Blackwell Publishing Inc; 1991;45: 853–853. 10.1111/j.1558-5646.1991.tb04354.x 28564058

[73] Gotthard K. Growth strategies of ectothermic animals in temperate environments In: Atkinson D, Thorndyke MC, editors. Animal Developmental Ecology. Oxford: Oxford: BIOS Scientific, 2001; 2001. pp. 1–18.

[74] Simons AM, Carrière Y, Roff DA. The quantitative genetics of growth in a field cricket. J Evol Biol. Blackwell Publishing Ltd; 1998;11: 721–721.

[75] Kvist J, Wheat CW, Kallioniemi E, Saastamoinen M, Hanski I, Frilander MJ. Temperature treatments during larval development reveal extensive heritable and plastic variation in gene expression and life history traits. Mol Ecol. 2013;22: 602–619. 10.1111/j.1365-294X.2012.05521.x 22429304

[76] Bolund E, Bouwhuis S, Pettay JE, Lummaa V. Divergent selection on, but no genetic conflict over, female and male timing and rate of reproduction in a human population. Proceedings of the Royal Society B: Biological Sciences. 2013;280: 20132002 10.1098/rspb.2013.2002 24107531 PMC3813330

[77] Siepielski AM, DiBattista JD, Carlson SM. It's about time: the temporal dynamics of phenotypic selection in the wild. Ecol Lett. England; 2009;12: 1261–1276. 10.1111/j.1461-0248.2009.01381.x 19740111

[78] Wilson K, Thomas MB, Blanford S, Doggett M, Simpson SJ, Moore SL. Coping with crowds: density-dependent disease resistance in desert locusts. Proceedings of the National Academy of Sciences. United States; 2002;99: 5471–5475. 10.1073/pnas.082461999 PMC122793 11960003

[79] Guttal V, Romanczuk P, Simpson SJ, Sword GA, Couzin ID. Cannibalism can drive the evolution of behavioural phase polyphenism in locusts. Liebhold A, editor. Ecol Lett. England; 2012;15: 1158–1166. 10.1111/j.1461-0248.2012.01840.x 22882379

[80] Haag CR, Saastamoinen M, Marden JH, Hanski I. A candidate locus for variation in dispersal rate in a butterfly metapopulation. Proceedings of the Royal Society B: Biological Sciences. The Royal Society; 2005;272: 2449–2456. 16271968 10.1098/rspb.2005.3235 PMC1599784

[81] Maeno K, Tanaka S. Phase-specific developmental and reproductive strategies in the desert locust. Bull Entomol Res. England; 2008;98: 527–534. 10.1017/S0007485308006044 18590599

[82] Maeno K, Gotoh T, Tanaka S. Phase-related morphological changes induced by [His]-corazonin in two species of locusts,Schistocerca gregariaandLocusta migratoria(Orthoptera: Acrididae). Bull Entomol Res. 2004;94: 349–357. 10.1079/BER2004310 15301700

[83] Gangwere SK, Chavin W, Evans FC. Methods of marking insects, with especial reference to Orthoptera (Sens. Lat.). Annals of the Entomological Society of America. 1964;57: 662–669. 10.1093/aesa/57.6.662

