# Supplementary material for: Extra Molting and Selection on Nymphal Growth in the Desert Locust
Source: PLoS One. 2016 May 26;11(5):e0155736. doi: 10.1371/journal.pone.0155736 (PMC4881952; doi:10.1371/journal.pone.0155736)
Supplement: S1 Table — For each trait, selected variables came from linear models displaying a ΔAIC < 2, among all possible models including a null model and a full model (i.e., containing all variables as well as every simple interaction between pairs of variables; see Methods). Note that for extra molting and for growth rate we used GLMs with logit link function and linear mixed model, respectively. We report estimates, standard deviations, t values (except for extra molting; #: z values) and P-values associated with each variable (and intercepts). For growth rate, L2, L3, L4 and L5 refer to 2nd to 5th nymphal instars. L3b refers to the added nymphal instar for individuals undergoing extra molting. Gray areas emphasize the outcome of models displaying the lowest AIC values. *** < 0.001 < ** < 0.01 < * < 0.05 <. < 0.1 (DOCX) [file pone.0155736.s001.docx]

| **Trait** | **AIC (ΔAIC)** | **Selected variables** | **Estimate** | **Std. error** | **t** | **P-value** | **Sig.** |
| --- | --- | --- | --- | --- | --- | --- | --- |
| **Extra molting #** | 109.68 (0) | (Intercept) | 4.506 | 1.142 | 3.947 | 7.910E-05 | *** |
|  |  | hatch weight | -0.390 | 0.094 | -4.153 | 3.280E-05 | *** |
|  |  | sex (male) | -2.104 | 0.563 | -3.739 | 1.850E-04 | *** |
|  | 111.14 (1.46) | (Intercept) | 5.2814 | 1.62 | 3.26 | 1.114E-03 | ** |
|  |  | hatch weight | -0.4562 | 0.1357 | -3.362 | 7.750E-04 | *** |
|  |  | sex (male) | -3.5821 | 2.1091 | -1.698 | 8.944E-02 | . |
|  |  | sex (male) : hatch weight | 0.1387 | 0.1885 | 0.736 | 4.618E-01 | NS |
| **Maximal nymphal weight** | 1337.52 (0) | (Intercept) | 1952.291 | 118.119 | 16.528 | < 2E-16 | *** |
|  |  | hatch weight | 20.083 | 8.864 | 2.266 | 2.569E-02 | * |
|  |  | sex (male) | -533.119 | 134.029 | -3.978 | 1.340E-04 | *** |
|  |  | extra molting (yes) | 157.669 | 42.881 | 3.677 | 3.870E-04 | *** |
|  |  | hatch weight : sex (male) | -22.239 | 10.571 | -2.104 | 3.799E-02 | * |
|  | 1338.36 (0.84) | (Intercept) | 1908.951 | 125.073 | 15.263 | < 2E-16 | *** |
|  |  | hatch weight | 23.589 | 9.468 | 2.491 | 1.444E-02 |  |
|  |  | sex (male) | -522.822 | 134.318 | -3.892 | 1.830E-04 | *** |
|  |  | extra molting (yes) | 309.714 | 151.098 | 2.050 | 4.312E-02 | * |
|  |  | hatch weight : sex (male) | -23.255 | 10.610 | -2.192 | 3.081E-02 | * |
|  |  | hatch weight : extra molting (yes) | -14.406 | 13.728 | -1.049 | 2.966E-01 | NS |
|  | 1339.07 (1.55) | (Intercept) | 2209.910 | 29.240 | 75.587 | < 2E-16 | *** |
|  |  | sex (male) | -810.990 | 35.400 | -22.907 | < 2E-16 | *** |
|  |  | extra molting (yes) | 119.150 | 38.480 | 3.097 | 2.550E-03 | ** |
|  | 1339.13 (1.61) | (Intercept) | 1982.660 | 128.540 | 15.424 | < 2E-16 | *** |
|  |  | hatch weight | 18.300 | 9.360 | 1.955 | 5.347E-02 | . |
|  |  | sex (male) | -582.170 | 156.680 | -3.716 | 3.410E-04 | *** |
|  |  | extra molting (yes) | 137.970 | 53.790 | 2.565 | 1.187E-02 | * |
|  |  | hatch weight : sex (male) | -19.300 | 11.650 | -1.656 | 1.009E-01 | NS |
|  |  | sex (male) : extra molting (yes) | 54.650 | 89.600 | 0.610 | 5.434E-01 | NS |
|  | 1339.13 (1.61) | (Intercept) | 2226.460 | 31.510 | 70.659 | < 2E-16 | *** |
|  |  | sex (male) | -838.320 | 40.490 | -20.705 | < 2E-16 | *** |
|  |  | extra molting (yes) | 83.290 | 46.380 | 1.796 | 7.560E-02 | . |
|  |  | sex (male) : extra molting (yes) | 112.860 | 82.280 | 1.372 | 1.733E-01 | NS |
| **Development time** | 327.37 (0) | (Intercept) | 21.828 | 0.620 | 35.205 | < 2E-16 | *** |
|  |  | hatch weight | -0.041 | 0.044 | -0.935 | 3.52E-01 | NS |
|  |  | sex (male) | 0.281 | 0.284 | 0.989 | 3.251E-01 | NS |
|  |  | extra molting (yes) | 1.933 | 1.133 | 1.706 | 9.120E-02 | . |
|  |  | hatch weight : extra molting (yes) | 0.253 | 0.099 | 2.561 | 1.2E-02 | * |
|  |  | sex (male) : extra molting (yes) | 0.932 | 0.588 | 1.584 | 1.165E-01 | NS |
|  | 327.59 (0.22) | (Intercept) | 22.826 | 0.988 | 23.096 | < 2E-16 | *** |
|  |  | hatch weight | -0.116 | 0.072 | -1.598 | 1.133E-01 | NS |
|  |  | sex (male) | -1.124 | 1.123 | -1.001 | 3.192E-01 | NS |
|  |  | extra molting (yes) | 1.425 | 1.195 | 1.192 | 2.362E-01 |  |
|  |  | hatch weight : extra molting (yes) | 0.281 | 0.101 | 2.785 | 6.460E-03 | ** |
|  |  | sex (male) : extra molting (yes) | 1.312 | 0.656 | 2.001 | 4.824E-02 | * |
|  |  | hatch weight : sex (male) | 0.108 | 0.083 | 1.294 | 1.989E-01 | NS |
|  | 328 (0.63) | (Intercept) | 21.636 | 0.613 | 35.310 | < 2E-16 | *** |
|  |  | hatch weight | -0.036 | 0.044 | -0.822 | 4.129E-01 | NS |
|  |  | sex (male) | 0.499 | 0.251 | 1.989 | 4.950E-02 | * |
|  |  | extra molting (yes) | 2.600 | 1.060 | 2.454 | 1.590E-02 | * |
|  |  | hatch weight : extra molting (yes) | 0.217 | 0.097 | 2.239 | 2.740E-02 | * |
|  | 329.172 | (Intercept) | 21.201 | 0.207 | 102.420 | < 2E-16 | *** |
|  |  | sex (male) | 0.459 | 0.251 | 1.833 | 6.990E-02 | . |
|  |  | extra molting (yes) | 4.857 | 0.272 | 17.828 | < 2E-16 | *** |
| **Growth rate** | -1400.72 (0) | (Intercept) | 0.529 | 0.027 | 19.588 | < 2E-16 | *** |
|  |  | L2 | -0.125 | 0.033 | -3.817 | 1.550E-04 | *** |
|  |  | L3 | -0.131 | 0.033 | -4.013 | 7.060E-05 | *** |
|  |  | L3b | -0.220 | 0.062 | -3.571 | 3.930E-04 | *** |
|  |  | L4 | -0.224 | 0.033 | -6.853 | 2.480E-11 | *** |
|  |  | L5 | -0.314 | 0.033 | -9.601 | < 2E-16 | *** |
|  |  | extra molting (yes) | 0.019 | 0.030 | 0.635 | 5.265E-01 | NS |
|  |  | hatch weight | -0.013 | 0.002 | -6.324 | 5.980E-10 | *** |
|  |  | sex (male) | -0.030 | 0.007 | -4.269 | 4.400E-05 | *** |
|  |  | hatch weight : extra molting (yes) | -0.006 | 0.003 | -2.030 | 4.477E-02 | * |
|  |  | L2 : hatch weight | 0.011 | 0.003 | 4.221 | 2.970E-05 | *** |
|  |  | L3 : hatch weight | 0.010 | 0.003 | 3.854 | 1.340E-04 | *** |
|  |  | L3b : hatch weight | 0.016 | 0.006 | 2.714 | 6.910E-03 | ** |
|  |  | L4 : hatch weight | 0.013 | 0.003 | 5.027 | 7.310E-07 | *** |
|  |  | L5 : hatch weight | 0.015 | 0.003 | 5.482 | 7.150E-08 | *** |
|  | -1398.91 (1.81) | (Intercept) | 0.530 | 0.027 | 19.481 | < 2E-16 | *** |
|  |  | L2 | -0.125 | 0.033 | -3.817 | 1.550E-04 | *** |
|  |  | L3 | -0.131 | 0.033 | -4.013 | 7.060E-05 | *** |
|  |  | L3b | -0.220 | 0.062 | -3.558 | 4.130E-04 | *** |
|  |  | L4 | -0.224 | 0.033 | -6.853 | 2.480E-11 | *** |
|  |  | L5 | -0.314 | 0.033 | -9.601 | < 2E-16 | *** |
|  |  | extra molting (yes) | 0.014 | 0.032 | 0.436 | 6.637E-01 | NS |
|  |  | hatch weight | -0.013 | 0.002 | -6.339 | < 2E-16 | *** |
|  |  | sex (male) | -0.032 | 0.008 | -3.910 | < 2E-16 | *** |
|  |  | hatch weight : extra molting (yes) | -0.005 | 0.003 | -1.887 | 6.181E-02 | . |
|  |  | L2 : hatch weight | 0.011 | 0.003 | 4.221 | 2.970E-05 | *** |
|  |  | L3 : hatch weight | 0.010 | 0.003 | 3.854 | 1.340E-04 | *** |
|  |  | L3b : hatch weight | 0.016 | 0.006 | 2.700 | 7.201E-03 | ** |
|  |  | L4 : hatch weight | 0.013 | 0.003 | 5.027 | 7.310E-07 | *** |
|  |  | L5 : hatch weight | 0.015 | 0.003 | 5.482 | 7.150E-08 | *** |
|  |  | sex (male) : extra molting (yes) | 0.007 | 0.016 | 0.436 | 6.638E-01 | NS |
|  | -1398.78 (1.94) | (Intercept) | 0.524 | 0.032 | 16.222 | < 2E-16 | *** |
|  |  | L2 | -0.125 | 0.033 | -3.816 | 1.550E-04 | *** |
|  |  | L3 | -0.131 | 0.033 | -4.013 | 7.060E-05 | *** |
|  |  | L3b | -0.220 | 0.062 | -3.558 | 4.130E-04 | *** |
|  |  | L4 | -0.225 | 0.033 | -6.853 | 2.480E-11 | *** |
|  |  | L5 | -0.315 | 0.033 | -9.601 | < 2E-16 | *** |
|  |  | extra molting (yes) | 0.020 | 0.030 | 0.665 | 5.073E-01 | NS |
|  |  | hatch weight | -0.013 | 0.003 | -5.090 | 6.430E-07 | *** |
|  |  | sex (male) | -0.024 | 0.026 | -0.893 | 3.741E-01 | NS |
|  |  | hatch weight : extra molting (yes) | -0.006 | 0.003 | -2.045 | 0.043 | * |
|  |  | L2 : hatch weight | 0.011 | 0.003 | 4.221 | 2.970E-05 | *** |
|  |  | L3 : hatch weight | 0.010 | 0.003 | 3.854 | 1.340E-04 | *** |
|  |  | L3b : hatch weight | 0.016 | 0.006 | 2.701 | 7.172E-03 | ** |
|  |  | L4 : hatch weight | 0.013 | 0.003 | 5.027 | 7.310E-07 | *** |
|  |  | L5 : hatch weight | 0.015 | 0.003 | 5.482 | 7.150E-08 | *** |
|  |  | hatch weight : sex (male) | -0.001 | 0.002 | -0.246 | 8.063E-01 | NS |
